# Characterization of Patient Interest in Provider-Based Consumer Health Information Technology: Survey Study

**DOI:** 10.2196/jmir.7766

**Published:** 2018-04-19

**Authors:** Joseph Featherall, Brittany Lapin, Alexander Chaitoff, Sonia A Havele, Nicolas Thompson, Irene Katzan

**Affiliations:** ^1^ Cleveland Clinic Lerner College of Medicine Cleveland Clinic Cleveland, OH United States; ^2^ Neurological Institute Center for Outcomes Research & Evaluation Cleveland Clinic Cleveland, OH United States; ^3^ Lerner Research Institute Department of Quantitative Health Sciences Cleveland Clinic Cleveland, OH United States; ^4^ School of Medicine Case Western Reserve University Cleveland, OH United States

**Keywords:** consumer health informatics, medical informatics, self efficacy, self-management, telemedicine, patient-centered care

## Abstract

**Background:**

Consumer health information technology can improve patient engagement in their health care and assist in navigating the complexities of health care delivery. However, the consumer health information technology offerings of health systems are often driven by provider rather than patient perspectives and inadequately address patient needs, thus limiting their adoption by patients. Consideration given to patients as stakeholders in the development of such technologies may improve adoption, efficacy, and consumer health information technology resource allocation.

**Objective:**

The aims of this paper were to measure patient interest in different health system consumer health information technology apps and determine the influence of patient characteristics on consumer health information technology interest.

**Methods:**

Patients seen at the Cleveland Clinic Neurological Institute were electronically surveyed on their interest in using different consumer health information technology apps. A self-efficacy scale, Patient Health Questionnaire-9 depression screen, and EuroQol 5 dimensions health-related quality of life scale were also completed by patients. Logistic regression was used to determine the influence of patient characteristics on interest in consumer health information technology in the categories of self-management, education, and communication.

**Results:**

The majority of 3852 patient respondents had an interest in all technology categories assessed in the survey. The highest interest was in apps that allow patients to ask questions of providers (3476/3852, 90.24%) and to schedule appointments (3211/3839, 83.64%). Patient interest in consumer health information technology was significantly associated with greater depression symptoms, worse quality of life, greater health self-efficacy, and smartphone ownership (*P*<.001 for all listed).

**Conclusions:**

Patients should be viewed as active stakeholders in consumer health information technology development and their perspectives should consistently guide development efforts. Health systems should consider focusing on consumer health information technologies that assist patients in scheduling appointments and asking questions of providers. Patients with depression should also be considered for targeted consumer health information technology implementation. Health self-efficacy is a valid predictor of consumer health information technology interest and may play a role in the utilization of consumer health information technologies. Health systems, broadly, should put forth greater effort to understand the needs and interests of patients in the consumer health information technology development process. Consumer health information technology design and implementation may be improved by understanding which technologies patients want.

## Introduction

### Background

Health care is undergoing enormous transformation in pursuit of achieving the triple aim: enhanced individual experience of care, improved health of populations, and reduced per capita costs of care [1,2]. Consumer health information technology (CHIT) has been defined in a number of ways [3-5] but has recently been well described by Tao et al as consumer-centered electronic tools, technologies, apps, or systems that are interacted with directly by health consumers to provide them with data, information, recommendations, or services for promotion of health and health care [6]. Such technologies are a key component of the patient-centered transformation of health care systems [5,7,8].

Implementation of CHIT is often driven by provider perspectives and available technologies rather than by the examined needs and motivations of users [5,9]. Of the largest 100 US hospitals, 66 provide mobile apps, but only 2% of patients at these hospitals are using them—an indication that health systems inadequately address the interests of patients in the CHIT design process [10]. Despite benefits in transparency and access associated with freely allowing patients to view their health data, health systems provide only limited data [11]. Focus groups have demonstrated that patients have innovative and useful perspectives as to how CHIT can improve health and ease patient workload [12-14]. Consumer perception of benefit, convenience, and integration into daily life is necessary for the successful implementation of CHIT and should be incorporated into the CHIT development process [4,15]. CHIT has been shown to improve health outcomes, but successful implementation requires that the needs of patients, that is, end users, are adequately met [3]. Overall, there is increasing recognition that health systems should systematically gather patient perspectives and use them to inform the design of CHIT [16].

The adoption of CHIT is influenced by a number of patient characteristics, but further investigation is necessary to fully characterize the CHIT needs of specific patient populations for optimum engagement and adoption [17]. For example, previous explorations have found patient portal adoption is associated with ethnic, educational, and cultural factors [18]; digital health is not used by the fastest-growing age segment of the US population, the elderly [19]; and patients who are sicker are more likely to search the internet for information and use this information during a provider visit [20]. However, these past studies have been limited in their scope, often exploring only how consumers react to already existing single CHIT solutions and not investigating the broad range of technology interests of patients. Further understanding of the technologies that patients want and the characteristics of these patients can prospectively guide strategies for providers and health systems investing in CHIT development.

Broadly, provider-based CHITs can be classified into one of 3 categories: patient-provider communications, education, and self-management technologies. Patient-provider communication technologies, including telecare, secure messaging, Web visits, and scheduling apps have demonstrated promise [21-25]. These apps improve patient convenience in accessing their health care teams. Recent data demonstrate that deficits in Web-based communication services may actually drive patients away from health systems [10]. Patient-centered prioritization of integrating communication technologies into the health system can be aided by quantifying and responding to the needs and interests of patients rather than basing technology development on data from historical technology adoption or qualitative data from small focus groups.

Electronic access to accurate patient educational resources is another established category of CHIT. Technologies that deliver educational programs to patients have demonstrated improvements in diverse outcomes, such as medication compliance [26,27], preprocedure anxiety (iPod-based modules) [28], postoperative perception of pain (educational website) [29], and plasma cholesterol (computer-based dietary workbook) [30]. During 2012, 59% of Americans searched for health-related information on the Web [31]; a smaller, more recent study indicates that internet health information seeking is global and likely increasing [32]. The use of electronic education tools in patient care varies according to patient access to the internet and patient awareness of digital education resources [33,34]. Knowledge of other patient factors that impact the use of educational resources can help design, development, and implementation strategies for education-related CHIT.

Self-management technologies encompass a third category of CHITs and have been shown to improve outcomes and reduce cost [35,36]. These include reminder systems, physical activity trackers, physiologic data (such as blood pressure or glucose) monitors, and apps that record self-reported health status over time. Despite the growing literature on this subject, limited information currently exists on how these technologies should be designed and which patients desire such programs [37-39]. Moreover, whether or not patients desire these technologies to be offered by their health care providers, as compared with third party CHIT offerings, has yet to be explored.

In addition to gaps in the literature with regard to specific patient characteristics associated with preference for each classification of CHIT, guidance on which types of CHIT apps are more likely to be adopted by patients is not well established [40]. Despite the “perceived utility” of a CHIT being a very reliable predictor of acceptance of technology, the utility of the technology to patients is addressed in very few studies [3]. There have been survey studies regarding the factors driving acceptance of CHIT; however, these studies investigate why patients choose to accept a single technology and did not ask, “In which technologies do patients have interest [39,41-43]?”

### Objectives

The aims of this study are as follows: (1) to quantify patients’ interests in CHIT by surveying patients seen in the ambulatory clinics of the Cleveland Clinic Neurological Institute and (2) to examine the influence of patient demographics, health-related quality of life, health self-efficacy, and depression on their interest in 3 categories of CHIT: patient-provider communication, educational resources, and self-management tools. This information can be used to better target the development and implementation of provider-based CHIT apps of greatest importance to patients.

## Methods

### Survey Design

The CHIT interest survey was designed to cover technologies that are common candidates for offerings by health systems. The technologies were grouped into 3 main categories: education, communication with health care providers, and self-management. Questions were developed by a multidisciplinary team of clinicians, researchers, and patients. Questions were evaluated by the team for content, ease of understanding, usefulness, and comprehensiveness in an iterative process and reviewed by the Neurological Institute Patient Advisory Committee for content and clarity [44]. The survey asked patients to rank their interest in various CHIT apps using a Likert scale with the following measurements: “not at all interested” (0), “not very interested” (1), “neutral” (3), “somewhat interested” (3), and “very interested” (4) (Multimedia Appendix 1). The survey was piloted, and response distributions were evaluated by the team to ensure no significant ceiling or floor effects. Internal reliability was assessed by Cronbach alpha.

To determine the impact of patient self-efficacy and current use of technology on interest in CHIT, the survey included a validated 5-item health self-efficacy questionnaire developed by Lee et al [45], as well as questions on current use of smartphones and tablet computers. The self-efficacy questionnaire included the following statements: (1) “I am confident I can have a positive effect on my health,” (2) “I have set some definite goals to improve my health,” (3) “I have been able to meet the goals I set for myself to improve my health,” (4) “I am actively working to improve my health,” and (5) “I feel that I am in control of how and what I learn about my health.” Patients rated their level of agreement with these statements using a Likert scale with the following measurements: “disagree very much”(0), “disagree”(1), “neutral”(2), “agree”(3), and “agree very much”(4). The health self-efficacy score was calculated by summing the responses to the 5 questions; thus, scores of “0” and “20” indicate the lowest and highest possible health self-efficacy scores, respectively.

### Patient Selection and Data Collection

Through the Knowledge Program patient-entered data collection system, patients complete the electronic questionnaires before their visits to the ambulatory clinics of the 3 pediatric and 14 adult condition–based centers of the Cleveland Clinic Neurological Institute [46]. These centers manage patients with neurological, neurosurgical, rehabilitation, and mental health conditions. Patients were given the option to complete the questionnaires through a MyChart patient portal (Epic, Epic Systems, Verona, Wisconsin) up to 5 days before their visits. Patients not completing questionnaires beforehand completed them using tablet computers in the clinic lobby at the time of check-in for their appointment. In addition to disease-specific instruments, all patients completed the EuroQol 5 Dimensions (EQ-5D) and Patient Health Questionnaire-9 (PHQ-9) as part of their regular questionnaire set. The EQ-5D is a commonly used 5-item generic scale that measures health-related quality of life [47]. Scores are transformed into utility weights derived from the general population and range from −0.109 (state worse than death) to 1.00 (best possible health). The PHQ-9 is a 9-item depression screen that ranges from 0 to 27, with higher scores indicating more depressive symptoms [48].

Between January 2013 and December 2015, patients seen in the 14 adult clinics who completed their questionnaires through MyChart were asked to complete the CHIT survey at the end of their regular questionnaire set. The CHIT survey was administered in each center until completed by at least 200 patients per center. As patient volume is variable across centers, a minimum response from 200 patients per center was established to ensure an adequate representation of patients. The survey was administered primarily through MyChart to avoid workflow disruptions at the time of the visit. To assess potential bias introduced by the survey delivery method, additional surveys were completed by patients seen in the Spine Center in the clinic lobby using tablet computers. Patient demographics and selected comorbid conditions (diabetes, atrial fibrillation, cancer, asthma, depression, congestive heart failure, coronary artery disease, chronic renal insufficiency, and hypertension) were identified from encounter diagnoses, problem list, or medical history sections of the electronic health record. Approximate household income was estimated based on 2010 census data by zip code. Patients 18 years of age and younger were excluded from the study. The study was approved by the Cleveland Clinic Institutional Review Board.

### Statistical Analysis

Individual survey item responses were summarized using frequency count with percentage. Internal reliability of self-efficacy and the CHIT survey were assessed using Cronbach alpha. Patient characteristics are presented as mean with SD or median with interquartile range for continuous variables, and frequency count with percentage for categorical variables. For the purpose of developing a predictive model, potential uses of CHIT were organized into 3 categories: education (educational offerings and online discussion forum), communication with providers (booking appointments, asking provider questions, and entering medical history), and self-management (reminder systems, tracking physiologic data, tracking physical activity, recording lifestyle information, comparing health to others, and tracking health status information). Given the distribution of patient response, with very few patients indicating “not at all interested,” high interest was defined by the multidisciplinary team as patients answering “very interested” to one or more questions within a category, whereas patients not responding with at least one “very interested” in the category were defined as low interest. A sensitivity analysis was conducted to define high-interest in a category as a response of “very interested” or “somewhat interested” to every question within that category. Low-interest for the sensitivity analysis was defined as a response of “neutral,” “not very interested,” or “not at all interested” to every question within the category.

Logistic regression models were constructed to determine univariate predictors of interest in the 3 CHIT domains. Independent predictors of the 3 outcomes were assessed using multivariable logistic regression models. Due to issues of multicollinearity, an indicator variable for any comorbidity (excluding depression) was included in the multivariable models in lieu of specific comorbidities, and smartphone ownership was included over tablet ownership as determined a priori. All potential interactions were evaluated within the final models at *P*<.05.

A subset analysis was conducted to assess potential bias introduced by surveying patients via the MyChart patient medical record portal. The 3 CHIT domain outcomes as well as individual question mean Likert scores for Spine Center patients completing the survey via MyChart were compared with those of Spine Center patients completing the survey in the lobby of the clinic using chi-squared test and Mann-Whitney *U* test, respectively.

Additionally, univariate and multivariable logistic regression models for the 3 outcomes were constructed as described above within Spine Center patients, including the location of survey completion as a covariate, in order to test for completion location influence on patient interest. Statistical significance was established throughout at an alpha level of .05. All statistical analyses were conducted using SAS version 9.4 (SAS Institute Inc, Cary, NC).

## Results

### Patient Characteristics

The study cohort consisted of 3852 patients who completed a survey: 3735 in MyChart and 117 in the Spine Center lobby. The mean age of the study cohort was 57.0 (SD 14.8) years (Table 1). The majority of the cohort was white (3115/3353, 92.90%), female (2351/3852, 61.03%), and married (2204/3351, 65.77%). Respondents had an average of 2 comorbid conditions, with the most common being hypertension (1465/2615, 56.02%). The majority of patients reported owning a smartphone (2640/3799, 69.49%) and a tablet computer (2103/3809, 55.21%).

**Table 1:** Clinical characteristics of the study cohort (N=3852).

Demographics		Value
Age (years), mean (SD)		57.0 (14.8)
Female, n (%)		2351 (61.03)
**Race (N=3353), n (%)**		
	White	3115 (92.90)
	African American	196 (5.85)
	Other	42 (1.25)
**Marital status (N=3351), n (%)**		
	Married	2204 (65.77)
	Single	748 (22.32)
	Divorced/widowed	399 (11.91)
Household income (in US $)^a^, N=3326, median (Q1-Q3)^b^		54,578 (44,371-66,360)
Any comorbid condition, N=2615, n (%)		2087 (79.81)
Number of comorbidities^c^, median (Q1-Q3)		2 (1-3)
**Health status, median (Q1-Q3)**		
	PHQ-9^d^, N=3434	6 (2-11)
	EQ-5D^e^, N=3483	0.78 (0.60-0.83)
	Health efficacy score, N=3791	15 (12-17)
**Personal mobile devices, n (%)**		
	Smartphone ownership (N=3799)	2640 (69.49)
	Tablet ownership (N=3809)	2103 (55.21)

^a^Median household income by zip code.

^b^Q1: first quartile, Q3: third quartile.

^c^Comorbid conditions include diabetes, atrial fibrillation, cancer, coronary artery disease, chronic renal insufficiency, and hypertension.

^d^PHQ-9: Patient Health Questionnaire-9.

^e^EQ-5D: EuroQol 5 Dimensions.

### Health Self-Efficacy

Internal reliability of this scale was high (Cronbach alpha=.845). The majority of patients responded affirmatively to all self-efficacy items, except the ability to meet health goals. Regarding meeting health goals, only 1663 out of 3815 (43.59%) respondents indicated that they agreed with the health efficacy statement. Regarding ability to have a positive effect on one’s health, 3274 out of 3825 (85.59%) patients indicated perceived self-efficacy in this area (1597/3825, 41.75%, “Agree” and 1677/3825, 43.84%, “Agree very much”).

### Consumer Health Information Technology Interest

**Figure 1:**
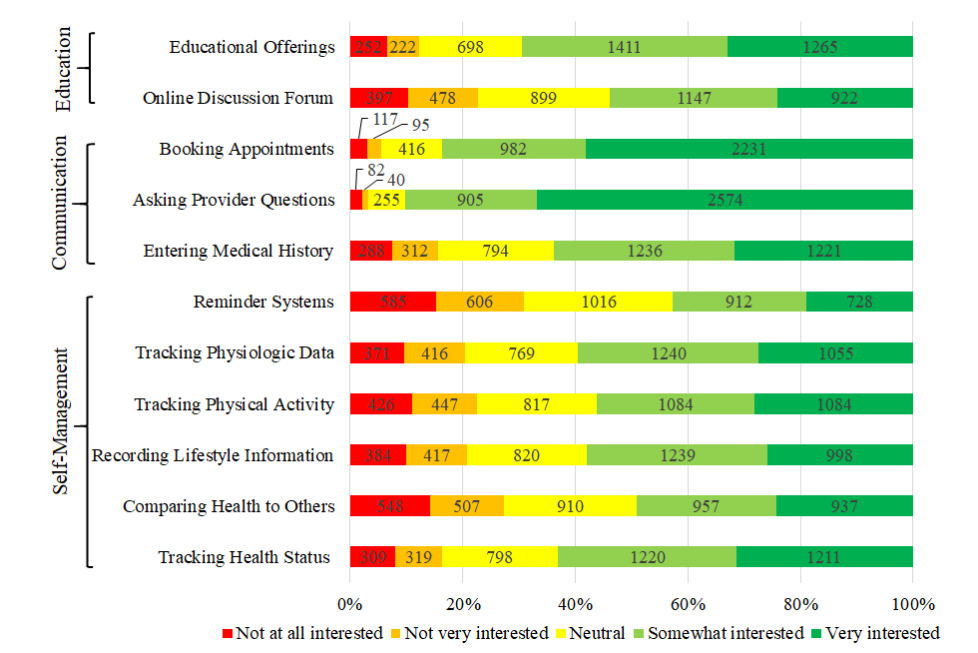
Response distribution to consumer health information technology interest survey questions. Response distributions (n) are shown for the level of interest to each question on the health information technology survey. Questions are grouped by category: education, communication, and self-management.

Internal reliability of the CHIT survey was good, with Cronbach alpha of .832 for education, .762 for self-management, and .898 for communication. The majority of patients indicated interest in all of the CHIT categories asked in the survey (Figure 1). Patients expressed the greatest interest in the communication category, specifically booking appointments (2227/3839, 58.01%, “very interested” and 984/3839, 25.63%, “interested”) and asking their provider questions (2570/3852, 66.72%, “very interested” and 906/3852, 23.52%, “interested”).

There were no categories in which a majority of subjects reported being disinterested in a service potentially offered by health technologies. However, greater than half of subjects reported neutral interest or less when asked about their interest in a technology that acted as a reminder system (2207/3845, 57.40%) or that would allow subjects to compare their health with others (1958/3848, 50.88%).

The majority of patients (2729/3838, 71.10%) were very interested in one of the items comprising the domain of communication, with 50.50% (1938/3838) being very interested in the domain of self-management and over a third of patients (1362/3843, 33.44%) being very interested in one of the items comprising the domain of education.

In the unadjusted analysis, patients were significantly more likely to be “very interested” in all 3 domains if they were female, African American (as compared with Caucasian), single (as compared with married), owned a smartphone, and had higher depression and health self-efficacy scores. They were less likely to be “very interested” in CHIT if they were older, had better health-related quality-of-life, or if they had coronary artery disease or hypertension (data not shown).

After adjustment for patient characteristics, however, only higher depression ratings, higher health self-efficacy scores, and smartphone ownership remained significant predictors of patient interest in all 3 technology categories: education, communication, and self-management (Table 2). Health-related quality of life was also an independent predictor of lack of interest in education technologies (odds ratio [OR] 0.92, 95% CI 0.86-0.98 per 0.1 increase), and older age was an independent predictor of lack of interest in self-management technologies (OR 0.89, 95% CI 0.81-0.97 per decade increase). No interaction effects were found.

**Table 2:** Multivariable predictors of consumer health information technology (CHIT) outcomes: education, communication, and self-management.

Predictors		Education		Communication		Self-management	
		OR^a^ (95% CI)	*P* value	OR (95% CI)	*P* value	OR (95% CI)	*P* value
Age (per decade)		0.98 (0.89-1.07)	.60	0.98 (0.89-1.08)	.67	0.89 (0.81-0.97)	.01
Female Gender		0.97 (0.78-1.22)	.80	1.20 (0.96-1.50)	.12	0.99 (0.80-1.22)	.89
**Race**							
	White	Reference		Reference		Reference	
	African American	1.46 (0.96-2.22)	.08	1.12 (0.70-1.77)	.64	1.48 (0.97-2.26)	.07
	Other	1.48 (0.55-3.94)	.44	2.16 (0.61-7.65)	.23	1.33 (0.51-3.50)	.56
**Marital status**							
	Married	Reference		Reference		Reference	
	Single	1.12 (0.85-1.48)	.43	0.85 (0.64-1.41)	.28	1.02 (0.78-1.33)	.90
	Divorced/widowed	1.29 (0.95-1.76)	.11	0.80 (0.58-1.09)	.15	1.09 (0.81-1.46)	.58
Income (per US $10k)		0.99 (0.93-1.06)	.81	1.06 (0.99-1.13)	.09	1.04 (0.98-1.10)	.26
PHQ-9^b^		1.04 (1.01-1.06)	.003	1.04 (1.02-1.07)	<.001	1.05 (1.03-1.08)	<.001
EQ-5D^c^ Index (per 0.1)		0.92 (0.86-0.98)	.01	0.98 (0.92-1.05)	.58	0.97 (0.91-1.03)	.32
Health self-efficacy score		1.20 (1.16-1.25)	<.001	1.12 (1.08-1.16)	<.001	1.15 (1.11-1.19)	<.001
Smart phone ownership		1.83 (1.43-2.34)	<.001	1.75 (1.39-2.22)	<.001	1.77 (1.41-2.21)	<.001
Any comorbidity^d^		0.82 (0.62-1.07)	.14	1.10 (0.83-1.48)	.49	1.05 (0.81-1.37)	.72

^a^OR: odds ratio.

^b^PHQ-9: Patient Health Questionnaire-9.

^c^EQ-5D: Euroqol 5 Dimensions.

^d^Comorbid conditions include diabetes, atrial fibrillation, cancer, coronary artery disease, chronic renal insufficiency, and hypertension.

### Sensitivity Analysis

Analyses were replicated after defining the outcome of interest in CHIT categories as a response of “somewhat interested” or “very interested” for every question within each category. Using this definition, 50.42% (1938/3843) of patients expressed interest in the education questions, 58.52% (2246/3838) in the communication questions, and 22.00% (844/3838) in the self-management questions. The independent predictors of interest in education and self-management remained the same as those presented in Table 2. Predictors for communication also remained the same, with the addition of decreasing age (OR 0.89, 95% CI 0.81-0.97 per decade increase).

### Subset Analysis

To assess whether completion location biased the study results, 117 out of 904 (12.9%) patients seen in the Spine Center completed the technology survey in the lobby, and results were compared with 787 out of 904 (87.1%) patients who completed the survey using MyChart before their Spine Center visit. In the unadjusted analysis, there were no significant differences in interest in CHIT regarding education or self-management (data not shown). Regarding communication questions, 513 of the 787 patients (65.2%) who completed the survey via MyChart indicated they were “very interested” in asking their provider questions compared with 64 of the 117 (54.7%) waiting room responders (*P*=.03). Similarly, 441 of 782 (56.4%) MyChart respondents expressed they were “very interested” in booking appointments versus 50 of the 117 (42.7%) patients in the waiting room (*P*=.006). After adjustment for patient characteristics, survey completion through MyChart was not a significant predictor of interest in communication (OR 1.81, 95% CI 0.85-3.85; *P*=.12).

## Discussion

### Principal Findings

CHIT is a promising strategy to enhance individual experience of care and improve the health of populations. CHIT has been demonstrated to engage patients, enhance clinical interventions, and improve health outcomes [49,50]. Focusing on CHIT approaches that are of greatest interest to patients would allow better allocation of resources by health care institutions that are struggling to contain costs and provide superior care in the current environment of reducing reimbursements. Our study found that over half of patient respondents are interested in CHIT apps that provide education, methods to communicate with healthcare providers, and self-management tools. The greatest interest is in CHIT apps that allow patients to ask questions of their health providers and to schedule appointments. CHIT apps that were of lowest interest to respondents were reminder systems and those that provided the ability to compare health status with others with the same condition.

Historically, the design of CHIT has been largely guided by provider attributes, financial incentives, and provider’s perceptions of patient needs rather than the needs and interests of patients themselves [5,17,51-53]. While a recent global survey indicated that only 8% of hospital-produced mobile apps offer the ability to book appointments [10], this study indicates that 83.65% (3213/3841) of current health system patients are interested in this functionality (Figure 1). Current estimates indicate that 7% of patients have changed their health systems because of deficits in online services [10]. This study is currently the largest survey of patient interest in provider-based CHIT and addresses a broader range of technologies than any previous surveys, providing actionable information for health systems working to address the provider-based CHIT gap.

### Quality of Life (EQ-5D) and Education Interest

In this study, patients with worse health-related quality of life, as assessed by EQ-5D, had a greater interest in educational technologies than patients with better quality of life. This is consistent with prior literature that sicker patients are more likely to search for health information [20]. When targeting CHIT strategies for patients with poor quality of life, education-related CHIT apps may be especially beneficial.

### Depression (PHQ-9) and Overall Interest

Patients with increasingly severe depression, as assessed with the PHQ-9 scale, demonstrated increasing interest in all 3 CHIT categories: education, communication, and self-management. It has been established that patients with depression may prefer treatment that does not require face-to-face interaction [54]. Recent trials of mobile apps have also demonstrated efficacy in managing depression [55-57]. Although, in the past decade, there has been a sharp increase in depression and the depression CHIT market, the large majority of these apps are neither evidence-based nor affiliated with any established medical institution [58]. Depression, despite being a leading global cause of disability, remains underdiagnosed and undertreated [59]. The high interest of patients with depression in this study and the lack of widely available evidence-based CHITs for depression behoove providers to carefully consider this population for focused CHIT development efforts.

### Health Self-Efficacy and Overall Interest

General self-efficacy, the belief in one’s competence to cope with a broad range of stressful or challenging demands, correlates well with self-regulation, coping strategies, and well-being [60]. Health, nutrition, physical exercise, and alcohol resistance self-efficacies are valid predictors of constructive health-related behaviors [61-63]; health self-efficacy, specifically, has been shown to influence health information–seeking behaviors [45,64]. Perceived difficulty of use decreases patient acceptance of CHITs [3]. CHITs are often promoted as mechanisms for increasing patient “empowerment,” “engagement,” “activation,” and so on [65], but little data exist on the association between self-efficacy and interest in using CHIT [3]. Our data suggest that the underlying attitudes of patients toward their health influence the desire to use such technologies. Health self-efficacy may explain the underlying attitudes that assist patients in overcoming the learning curve required to adopt CHITs. Such a concept has practical implications in that such underlying attitudes may explain some of the variability in the efficacy of CHITs observed in trials within different populations [6]. Developers may also consider targeting high self-efficacy users for increasing the likelihood of a successful CHIT implementation.

### Smartphone Ownership and Overall Interest

Mobile health apps are poised to become a determining factor in restructuring old health care services and systems still based on physical relationships between patients and providers [66,67]. The data from this study indicate that smartphone use is a strong predictor of CHIT interest. Supporting this finding is a recent patient survey, in which 86% of respondents demonstrated interest in using a mobile app to improve their health [68]. Despite widespread enthusiasm and public interest, national survey data demonstrate that mobile apps offered by providers were lower rated by users and are not aligned with patient wants or needs [10]. Due to their ease of use, smartness, accessibility, mobility, and connectivity, smartphones are an effective and consumer-oriented technology platform; however, health systems consistently lag behind in mobile technology offerings [69]. These data, together, suggest that there exists a large opportunity for providers to improve their mobile offerings, focusing on usability and the functionalities patients desire.

### Limitations

This study has some limitations. The survey was administered to patients seen within the Cleveland Clinic Neurological Institute. The study sample was largely white (3115/3852, 92.90%), with a moderately high median annual household income (US $54,578); centers with different demographics may have different findings. However, these clinics see patients with a broad range of conditions, spanning from back pain to neurodegenerative disorders to psychiatric conditions. Moreover, this study assesses the CHIT interest of current health care users rather than the population-based surveys that are often used to assess attitudes and preferences, and thus, may more accurately reflect the preferences of current patients. Another limitation is the deployment of surveys via the MyChart patient portal, introducing possible responder bias as respondents using the patient portal may be more apt to use health technologies. However, there was no significant difference in patient interest in using CHIT between respondents receiving the survey via the patient portal and the sample of respondents completing the survey in the clinic lobby.

### Future Directions

This study provides insight into the interests and needs of current health system patients. This study also demonstrates how such information can be used to model patient interest to target technology offerings of providers. Future studies should include the development of more sophisticated predictive models; continuing to identify subpopulations defined by disease states, preferences, and need attributes will further optimize CHIT implementations.

### Conclusions

Health care is under pressure to improve outcomes and decrease cost; CHITs provide a promising solution. CHIT developers often pay minimal attention to patients’ motivations, needs, and psychosocial characteristics [70]. Such oversights may lead to underutilization of provider-based CHIT. In order to solve this problem, patients should be recognized as full stakeholders in health information systems and their perspectives should be systematically incorporated into development efforts [71]. Due to high patient interest, CHIT developers and health system clinician leaders should consider focusing CHIT development toward smartphone platforms and developing CHIT apps that allow patients to schedule appointments and to ask questions of their providers [72,73]. Patients with depression have a high interest in CHITs and thus may benefit from provider offerings of evidence-based CHITs. Health self-efficacy is an independent predictor of CHIT interest and should be considered in future attempts to explain CHIT adoption and efficacy patterns.

## Appendix

Multimedia Appendix 1 Consumer health information technology interest survey.

## Abbreviations

CHIT: consumer health information technology
EQ-5D: EuroQol 5 Dimensions
OR: odds ratio
PHQ-9: Patient Health Questionnaire-9
