# Supplementary material for: Characterization of Patient Interest in Provider-Based Consumer Health Information Technology: Survey Study
Source: J Med Internet Res. 2018 Apr 19;20(4):e128. doi: 10.2196/jmir.7766 (PMC6004033; doi:10.2196/jmir.7766)
Supplement: Multimedia Appendix 1 [file jmir_v20i4e128_app1.pdf]

How interested would you be in using each of the following electronic healthcare applications?

1. Recording and tracking self-reported *health status information* over time such as pain level, symptom level, or ability to perform daily activities
   - Very interested
   - Somewhat interested
   - Neutral
   - Not very interested
   - Not at all interested

2. Comparing your health status to others with a similar condition(s)
   - Very interested
   - Somewhat interested
   - Neutral
   - Not very interested
   - Not at all interested

3. Self-recording of *lifestyle* information over time such as sleep, diet, or medication adherence that can be tracked over time
   - Very interested
   - Somewhat interested
   - Neutral
   - Not very interested
   - Not at all interested

4. Tracking your physical activity data (such as step count) through a monitoring device
   - Very interested
   - Somewhat interested
   - Neutral
   - Not very interested
   - Not at all interested

5. Tracking your physiologic data (such as heart rhythm, weight, or blood pressure) through a monitoring device
   - Very interested
   - Somewhat interested
   - Neutral
   - Not very interested
   - Not at all interested

6. Entering your complete medical history (such as allergies, medical conditions, or prior surgical procedures) and social history (including family medical history, occupation, and tobacco use) through the patient personal health record for use during an upcoming visit.
   - Very interested
   - Somewhat interested
   - Neutral
   - Not very interested
   - Not at all interested

7. Ability to ask your provider questions
   - Very interested
   - Somewhat interested
   - Neutral
   - Not very interested
   - Not at all interested

8. Booking appointments with your provider
   - Very interested
   - Somewhat interested
   - Neutral
   - Not very interested
   - Not at all interested

9. Internet forum for online discussion of health topics that are tailored to your health condition
   - Very interested
   - Somewhat interested
   - Neutral
   - Not very interested
   - Not at all interested

10. Educational offerings tailored to your health condition(s)
   - Very interested
   - Somewhat interested
   - Neutral
   - Not very interested
   - Not at all interested

11. Having a reminder system to take your blood pressure or medication or to exercise
   - Very interested
   - Somewhat interested
   - Neutral
   - Not very interested
   - Not at all interested

*These questions comprise the CHIT survey only. These questions were deployed as a section of the complete survey, incorporating self-efficacy, PHQ-9, EQ-5D, etc.
